# Chimeric CD3ζ chains containing CD28 signalling motifs enhance antigen-specific IL-2 production and expansion of human TCR-engineered T cells *in vitro*

**DOI:** 10.1093/immadv/ltaf038

**Published:** 2026-04-01

**Authors:** Samuel J Burgess, Hans J Stauss, Emma C Morris

**Affiliations:** Institute of Immunity and Transplantation, Division of Infection and Immunity, University College London, London, United Kingdom; Institute of Immunity and Transplantation, Division of Infection and Immunity, University College London, London, United Kingdom; Institute of Immunity and Transplantation, Division of Infection and Immunity, University College London, London, United Kingdom

**Keywords:** T-cell receptor, TCR-T therapy, CD3-zeta, CD28, T-cell function

## Abstract

Gene-engineered T-cell products have been developed for immunotherapy to treat cancers, with great success observed in haematological malignancies but limited efficacy in treating solid cancers. TCR-engineered T cells utilize transferred TCRs targeting tumour-associated and cancer-specific peptides presented by MHC molecules. The CD3ζ chains are part of the TCR-CD3 complex expressed by T cells and mediate signal transduction when the TCR binds to MHC-presented peptides. In this study, we explored whether co-stimulation domains, that were effective in improving the function of T cells engineered with chimeric antigen receptors (CARs), can be exploited to improve the functionality of TCR-engineered T cells. We inserted the signalling domains of CD28 or 4–1BB at the membrane proximal or the membrane distal position of the intracellular tail of CD3ζ and engineered human T cells to express a specific TCR in combination with either modified CD3ζ or unmodified control. Antigen-specific *in vitro* stimulation assays revealed that T cells expressing CD3ζ constructs with CD28 signal domains displayed enhanced peptide-specific IL-2 production and, following repeated antigen stimulation, expanded to substantially greater numbers than T cells expressing unmodified CD3ζ. Importantly, greater expansion seen with the CD28-containing ζ did not result in any reduction of effector function as assessed by peptide-specific cytotoxicity and cytokine production. The data indicate that modification of the CD3ζ chain with a CD28 signal motif provides an opportunity to improve antigen-specific expansion and effector function of TCR-engineered T cells by combining signal 1 and co-stimulatory signal 2 in one molecular TCR-CD3 complex.

## Graphical Abstract

**Graphical Abstract:**
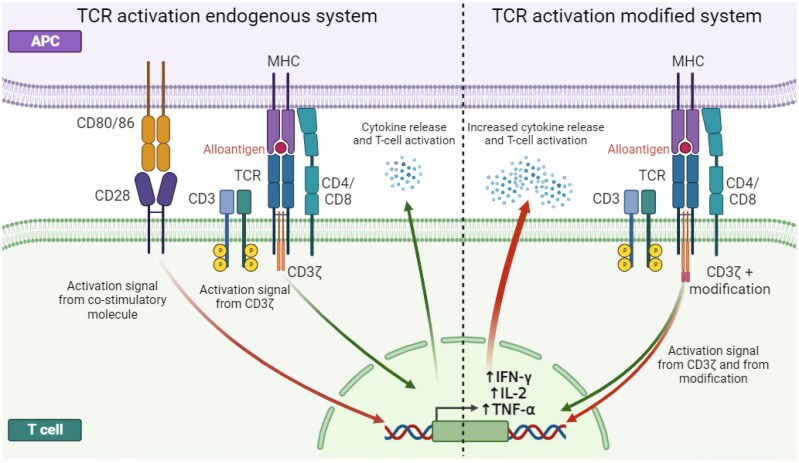

## Introduction

Gene-modified T-cell therapies, including chimeric antigen receptor T cells (CAR T cells) and T-cell receptor T cells (TCR T cells), are widely used in the treatment of haematological malignancies [1–4]. The antigen-specificity of cellular therapies can limit off-target toxicities compared to more conventional chemo- and radiotherapeutic approaches. Various CD19-targeting CAR T cells have established clinical efficacy in the treatment of acute lymphoblastic leukaemia, diffuse large B cell lymphoma, and mantle cell lymphoma, while BCMA CAR T cells are now widely used for multiple myeloma [5–7]. However, to date, both CAR T cells and TCR T cells have been less effective against solid tumours [6, 8, 9]. Unlike haematological malignancies, solid tumours can be associated with highly immunosuppressive tumour microenvironments. The presence of hypoxia, reduced nutrients, and immunosuppressive cells/cytokines limit T-cell functionality in the tumour microenvironment and promote T-cell exhaustion [10, 11].

For the treatment of solid tumours the utilization of TCR T cells, instead of CAR T cells, provides an exciting opportunity to target a wide range of both intracellular and cell surface antigens via MHC-presented peptides, whereas CAR T cells are restricted to cell surface antigens only. Current TCR T-cell therapies can target either tumour-associated antigens (TAA), such as NY-ESO1 and MAGE-A3, or tumour-specific antigen (TSA), such as KRAS G12D [12–14]. TCR T cells require a lower epitope density to activate the cells compared to CAR T cells, which is particularly useful as tumour cells can undergo antigen escape from downregulation of target antigen in response to treatments [15, 16].

Naturally, T cells are activated via their TCR [17, 18], which occurs when the TCR binds the peptide/MHC (p/MHC) complex on the surface of the target cell [17, 18]. The TCR signalling complex comprises six different molecules: TCR α chain, TCR β chain, CD3ε, CD3δ, CD3γ, and CD3ζ [19]. There are two CD3ζ molecules in the TCR complex, which are responsible for initiating downstream signalling from the TCR via domains called immunoreceptor tyrosine-based activation motifs (ITAMs) [20, 21]. Following TCR-p/MHC engagement, LCK phosphorylates the ITAMs, enabling the recruitment of ZAP70, which in turn activates LAT and then downstream signalling pathways leading to T-cell activation [20, 21]. Each CD3ζ has three ITAMs, which are the primary signalling component of the TCR and the rationale for their use as a key component in CAR constructs. TCR signalling is controlled by TCR affinity, the strength of the TCR-p/MHC interaction, and TCR avidity defined by the density of TCR molecules on the T-cell surface [22–24]. The readout of the T-cell response is functional avidity, which measures antigen-specific T-cell responses following stimulation with decreasing concentrations of peptide [22, 24].

Optimal T-cell activation requires signalling from the TCR (signal 1) combined with additional co-stimulation signals (signal 2). Common co-stimulation molecules include CD28, CD137 (41BB or TNFRSF9), and OX-40 [21, 25, 26]. CD28 binds to CD80/86 on the APCs, initiating phosphorylation of its signalling domains, SH2 and SH3, in turn allowing for downstream activation of transcription factors like NFκB, NFAT, and AP-1, along with initiating PI3K signalling [21, 26–28]. The activation of these molecules and respective signalling pathways primarily promotes increased IL-2 production but also improves T-cell proliferation and decreases apoptosis [29–33]. CD137 is part of the TNFR superfamily and binds to CD137L on the APCs, activating downstream signalling via TRAF1 and TRAF2 [34, 35]. This promotes T-cell survival and expansion, while also increasing IFNγ production [35–37].

To overcome the challenges facing TCR T-cell therapy, we aimed to exploit the lessons learnt from CAR T cells and equip TCRs with the ability to deliver both signal 1 and signal 2 following TCR-MHC engagement. By utilizing this additional signalling domain, we aim to enhance the functional output of the modified TCR T cells, promoting increased cytokine production and killing efficacy. To achieve this, we generated modified CD3ζ chains by introducing the signalling domains of the co-stimulation molecules CD28 and CD137. We found that the addition of a CD28 signalling domain to CD3z enhanced antigen-specific proliferation and IL-2 production of TCR-engineered T cells, while retaining the antigen-specific cytotoxic function.

## Methods

### Tissue culture and cell lines

All cell lines were grown at 37°C in 5% CO_2_ in a humidified atmosphere. Jurkat T cells and T2 cells were cultured in RPMI media (all from Life Technologies, Gibco), with 10% FCS, 1% penicillin/streptomycin, and 1% L-glutamine. Cells were passaged 1:4 every 48 hours to prevent overconfluence. HEK 293T cells were cultured in IMEM media (Merck), with 10% foetal calf serum (FCS), 1% penicillin/streptomycin (Invitrogen, 100 U/ml), and 1% L-glutamine (Gibco 2 mM). Cells were routinely detached using trypsin EDTA and passaged 1:4. Primary human T cells were cultured in TexMACS media (Miltenyi), with 1% penicillin/streptomycin and 100 U/ml IL-2 (Chiron). T cells were activated every 7–10 days with an activation cocktail including TexMACS media, with 200 U/ml IL-2 and 15μl/ml CD2/CD3/CD28 (StemCell). Human T cells were obtained from buffy coats supplied by NHSBT. CD3+ T cells were isolated from buffy coats using magnetic-associated cell sorting (MACS) (Miltenyi) as per the manufacturer's instructions.

### Plasmid generation

CD3ζ sequences and TCR sequences were ordered in string format from GeneArt. These sequences were ligated into the PDualb lentiviral vector backbone. Successful ligation was confirmed by restriction digests and DNA sequencing.

### Lentivirus generation and cell transduction

Lentivirus was generated using HEK293T packaging cells. HEK293T cells were plated at a concentration of 3–4 × 10^6^ cells/15 cm^2^ dish. After overnight culture, the media was replaced prior to transfection with two packaging vectors, p8.91 and VSVG, and the plasmid containing the CD3ζ DNA or the TCR DNA. For transfection, cells were suspended in Opti-MEM medium (Gibco) containing 40 μl/500 ml Fugene transfection reagent (Promega) and 2.5 μg p8.91, 1 μg VSVG, and 4 μg plasmid of interest were added. Following 20 minutes of incubation at room temperature (RT), the mixture was added to the HEK293T cells and incubated for a further 24 hours at 37°C and 5% CO_2_. After this, the media was changed and the cells were cultured for another 24 hours at 37°C and 5% CO_2_. Virus was isolated from the culture supernatant by ultracentrifugation (20 000 rpm, 2 hours, 4°C). The viral pellet was suspended in media and aliquoted. The virus was either used immediately or stored at −80°C.

Lentiviral titres were calculated using HEK293T cells. 2 × 10^5^ cells/well were plated and different dilutions of viral supernatant were added to each well, ranging from 1:100 to 1:100 000 (Virus: Media), prior to incubation for 48 hours at 37°C and 5% CO_2_. Following the incubation, the percentage of transduced cells was determined by detection of CD19 expression using flow cytometry.

Subsequent lentiviral transduction of Jurkat cells (5 × 10^5^ cells) or primary human T cells (1 × 10^6^ cells) were performed using an MOI of 1 for Jurkat cells and an MOI of five for primary human T cells. The cells were then incubated with the virus for 48 hours at 37°C and 5% CO_2_. The transduction efficiency was then analysed using flow cytometry.

### Flow cytometry and cell sorting

Cells were centrifuged at 1600 rpm for 5 minutes prior to removing culture media from the cell pellet. The pellet was re-suspended in 2%FCS/PBS containing the live/dead dye and cell surface antibodies required for the analysis and incubated at 4°C for 30 minutes. Following incubation, the cells were washed in an additional 2%FCS/PBS and centrifuged at 1600 rpm for 5 minutes, to remove any unattached antibodies. The cell pellets were then suspended in 2%FCS/PBS prior to analysis on the BD Fortessa or Cytek Aurora.

Intracellular cytokine staining was performed as above, but after the second wash, cells were suspended in fixation buffer (Invitrogen) for 20 minutes at 4°C. Following fixation, cells were washed in 2%FCS/PBS and re-suspended in 1× permeabilization buffer containing antibodies for intracellular cytokines and incubated for 60 minutes at 4°C. The cells were then washed using 2%FCS/PBS and centrifugation. The cells were then suspended in 2%FCS/PBS and analysed using the BD Fortessa or Cytek Aurora. Further flow cytometry analysis was then completed using FlowJo software V10. The antibodies used in this project were CD3 (PE-Cy7, BioLegend), TCR α/β (PE, BD Biosciences), mouse CD19 (efluor450, Invitrogen), CD8 (FITC, Invitrogen), CD28 (APC, Invitrogen), CD95 (BV711, BioLegend), CD45RO (PerCP-e710, Invitrogen), CCR7 (BUV737, Invitrogen), PD1 (BUV661, BD Biosciences), LAG3 (BUV805, Invitrogen), CD69 (APC, BD Biosciences), IL-2 (APC, BioLegend), IFNγ (PE, Invitrogen, 1:400), TNFα (BV421, BioLegend), CD3ζ (PerCP-e710, Invitrogen, 1:25), and Live/dead stain (APC-cy7, BioLegend, 1:1000). All antibodies were used at a 1:100 dilution unless otherwise stated.

### IL-2 enzyme linked immunosorbent assay (ELISA)

To perform the ELISA, Jurkat cells transduced with both the TCR and various CD3ζ constructs were co-cultured with T2 cells, which had been peptide pulsed with different concentrations of relevant peptide or with an irrelevant peptide. Control peptides used were the CMV-associated peptide, CMVpp65 (NLV), and the haemagglutinin peptide HA1. Cells were co-cultured in a 96-well plate, with 1 × 10^5^ Jurkat cells and 1 × 10^5^ T2 cells per well, with each condition set up in duplicate. The cells were cultured overnight at 37°C and 5% CO_2_. Concurrently, a 96-well ELISA plate was incubated overnight at 4°C with IL-2 capture antibody (1:250 dilution) suspended in 1× assay diluent (BioLegend). Following incubation, the plate was spun down at 1600 rpm for 5 minutes and the supernatant was removed. The ELISA plate was washed using 1× ELISA wash buffer (BioLegend), the same wash buffer was used for each wash step. The cell supernatant was added to the ELISA plate and incubated for 2 hours at RT, prior to plate washing. Then a mixture containing a detection antibody and streptavidin-HRP in 1× assay diluent was added to each well and incubated for 1 hour at RT. The plate was then washed. After this wash a 50:50 mixture of enzyme substrates (Substrate A and Substrate B (BD)) was added to each well and incubated for 30 minutes at RT away from light. After this incubation ELISA stop solution (BioLegend) was added to each well. The plate was immediately analysed using a plate reader (Thermo Scientific Multiskan FC) to obtain OD values.

### Short-term T-cell expansion cultures

7–10 days post-transduction, bulk CD3+ T cells were utilized in the expansion assay. Irradiated T2 stimulator cells (80 Gy) were peptide-loaded with 10 μM relevant peptide. Feeder cells (autologous PBMCs) were also irradiated (40 Gy). To set up the assay, 5 × 10^5^ T cells, 2 × 10^5^ T2 cells, and 2 × 10^6^ PBMCs were added to a well containing TexMACS media with 100 U/ml IL-2 and 5 ng/ml IL-7. The cells were then incubated for 7 days at 37°C with 5% CO_2_, and fresh media (without cytokine) were added every 2–3 days when needed. T cells were re-stimulated every 7 days, using the same method outlined above. Cells were kept in culture for up to 28 days.

### *In vitro* cytotoxicity assay

T cells were stimulated every 7 days and at each re-stimulation, an aliquot of T cells was removed for *in vitro* cytotoxicity assays. Relevant peptide-loaded T2 cells were labelled with 0.02 μM CFSE and irrelevant peptide-loaded T2 cells were labelled with 0.2 μM CFSE. Following 2 hours of peptide loading, the two different T2 cell populations were combined at a 1:1 ratio, with 5 × 10^4^ cells/well of each type. 1 × 10^5^ bulk transduced T cells were then added to each well and then incubated overnight. After incubation, killing of T2 cells was analysed using flow cytometry. The amount of antigen-specific cell killing was then calculated as:


$$\text{\%}\;Specific\;killing\;=\;100\;-\left[\begin{array}{c} \frac{\left(relevant/irrelevant\;T2\;cells\;with\;T\;cells\right)}{\left(relevant/irrelevant\;T2\;cells\;with\;no\;T\;cells\right)} \end{array}\right]\;x\;100.$$

### Cytokine production assay

As with the cytotoxicity assay, an aliquot of T cells was removed from the expansion assay and T2 cells were loaded with either relevant or irrelevant peptide. Following peptide loading, 1 × 10^5^ T2 cells containing either relevant or irrelevant peptide were added to individual wells. 1 × 10^5^ T cells were also added to each well. The cells were then cultured overnight in TexMACS media containing 5 μg/ml Brefeldin A. Following incubation, the cells were washed and then stained and analysed following the protocol described above.

### Statistical analysis

Data are represented as mean expression with ±SEM. Statistical tests were performed using GraphPad Prism (Version 10). TCR fold change is calculated from changes in MFI compared to the endogenous CD3ζ population. The statistical tests used to determine statistical significance were Student’s *t*-test and 2-way ANOVA with Dunnett's multiple comparison as indicated.

## Results

### CD3ζ with a CD28 signal domain enhances antigen-specific IL-2 secretion in human Jurkat T cells

**Figure 1:**
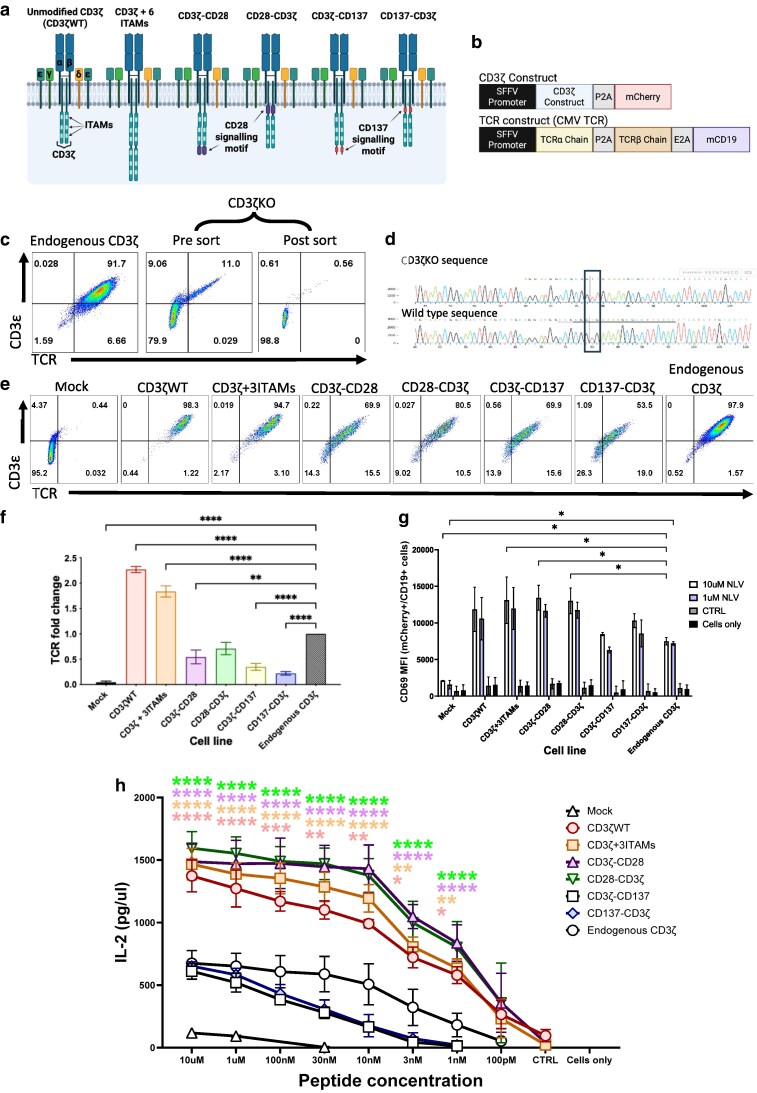
CD3ζ containing CD28 signalling motifs recover TCR expression following CD3ζKO and enhances cell activation and IL-2 secretion. (a) Schematic representation of the different CD3ζ modifications tested. CD3ζWT is unmodified CD3ζ. The dark blue represents the TCRα and TCRβ chains, the dark green the CD3ε, the green CD3δ, the yellow CD3γ, the light blue CD3ζ containing 3 ‘native’ ITAMs, the purple CD28 signalling domain, and the red CD137 signalling domain. (b) Vectors used in this project, the top vector contains the α and β chains of a CMV-specific TCR, followed by truncated murine CD19 (mCD19), the bottom vector contains the CD3ζ constructs followed by mCherry. (c) FACS plots demonstrating the KO of CD3ζ in the Jurkat cells. (d) The sorted cells for CD3ε−/TCR- were sent off for DNA sequencing to confirm the CD3ζKO on a DNA level. The electropherogram confirms this change with a one base pair deletion (Black box). (e) Flow cytometry plots showing the effects of the different CD3ζ modifications on TCR expression, identified by using CD3ε and TCR antibodies. The cells are from the mCherry+/mCD19+ or mCherry-/mCD19+ (TCR only) cell populations. (f) Graph depicting the difference in surface TCR expression, calculated using TCR MFI, in CD3ζKO Jurkat cells transduced with one of the different CD3ζ modifications compared to cells that express endogenous CD3ζ. (g) Graph summarizing the changes in CD69 MFI (CD69+ cells), following different peptide stimulations, 10 µM or 1 µM of relevant peptide (RP), 10 µM irrelevant peptide (IP) or cells only (no stimulation). (h) Graph shows the results from the IL-2 ELISA with cells stimulated with a range of peptide concentrations, IP, or no peptide (cells only). Significance values are compared to the endogenous CD3ζ population (*n* = 4). Statistical analysis was performed using the Student’s *t*-test (TCR expression) and the 2-way ANOVA (CD69 expression and IL-2 secretion) (*P < 0.05, **P < 0.01, ***P < 0.001, **** *P* < 0.0001). (a/b) Created in BioRender. Burgess, S. (2026) https://BioRender.com/0uz4xxj.

We tested whether the addition of CD28 or CD137 signalling motifs into the cytoplasmic tail of CD3ζ could equip the TCR–CD3 complex with improved function. We produced CD3ζ variants that contained CD28 or CD137 signalling domains that were inserted either proximal or distal to the native three ITAM motifs in the intracellular tail of the *ζ* chain (Fig. 1a). We also produced a CD3ζ construct with a duplication of the ‘native’ ITAM motifs, resulting in a construct with six ITAMs in total (Fig. 1a). Each of the CD3ζ variants were assembled in lentiviral vectors that contained mCherry, used as a marker to identify transduced cells (Fig. 1b).

We used human Jurkat 76 T cells, which express endogenous CD3 chains but no TCR, and introduced the α/β chains of a TCR-specific for a peptide epitope of human cytomegalovirus (CMV) with a vector that contained CD19 as a transduction marker (Fig. 1b). More than 90% of the CD19-positive Jurkat cells expressed high levels of the introduced TCR in combination with the CD3 complex, which was detected by staining with anti-CD3ε antibodies (Fig. 1c, left plot). We used CRISPR-Cas9 to disrupt the endogenous *CD3ζ* gene in these Jurkat cells, which resulted in loss of TCR and CD3 expression in 79.9% of cells (Fig. 1c, middle plot). FACS sorting was used to generate a Jurkat line with more than 98% of the cells lacking TCR/CD3 on the cell surface (Fig. 1c, right plot). Gene sequencing confirmed that the endogenous *CD3ζ* gene was successfully targeted by CRISPR, which resulted in a base pair insertion in exon 1, causing a frameshift in the open reading frame (Fig. 1d). These cells were then used to test whether the CD3ζ variants could reconstitute the surface expression of TCR/CD3 complexes, and whether they modified antigen-specific TCR function.

Figure 1e, f shows that all CD3ζ variants restored TCR/CD3 expression in transduced Jurkat cells, which have been gated on cells expressing the same level of mCherry+ and CD19+. However, TCR/CD3 surface expression was highest following transduction with unmodified wild-type CD3ζ and with the construct containing six ITAM motifs. In contrast, the CD3ζ containing CD28 or CD137 signalling motifs expressed significantly less TCR/CD3 on the cell surface, suggesting that the presence of these motifs reduced CD3ζ chain stability and/or its ability to efficiently assemble into TCR/CD3 complexes. Jurkat cells with an intact endogenous CD3ζ gene were used as controls for surface TCR/CD3 expression in these experiments (Fig. 1e, f).

Stimulation with TCR-recognized CMV peptide (NLV) demonstrated that wild-type CD3ζ and all variants mediated CD69 upregulation after stimulation with both 10 and 1 μM of the cognate peptide (NLV), but not after stimulation with an irrelevant control peptide (CTRL). Despite similar levels of peptide-specific CD69 upregulation observed between all constructs, they were all able to upregulate higher levels of CD69 compared to endogenous CD3ζ only cells (Fig. 1g). When examining the cell's ability to secrete IL-2, we observed significant differences between the different CD3ζ constructs (Fig. 1h). At all peptide concentrations, cells transduced with the CD28-containing constructs secreted at least twice as much IL-2 as cells with endogenous CD3ζ (Fig. 1h). The observation that TCR expression levels were lower in cells transduced with the CD28 constructs compared to cells with endogenous CD3ζ (Fig. 1f) indicates that the elevated antigen-specific IL-2 response is not caused by an increase in TCR avidity of the transduced Jurkat cells. Compared to Jurkat cells expressing endogenous CD3*ζ*, we also saw improved IL-2 secretion of cells transduced with either wild-type CD3ζ or the variant with 6 ITAMS (Fig. 1h), but in this case, the doubling of IL-2 secretion correlated with doubling of TCR expression levels (Fig. 1f), suggesting that increased TCR avidity of transduced Jurkat cells resulted in improved IL-2 secretion. The reduced IL-2 secretion by cells transduced with the CD3ζ constructs containing CD137 signalling motifs correlated with reduced TCR expression compared to Jurkat cells with endogenous CD3ζ, indicating that, unlike CD28, the CD137 motifs were unable to rescue IL-2 production of ‘TCR low’ cells.

**Figure 2:**
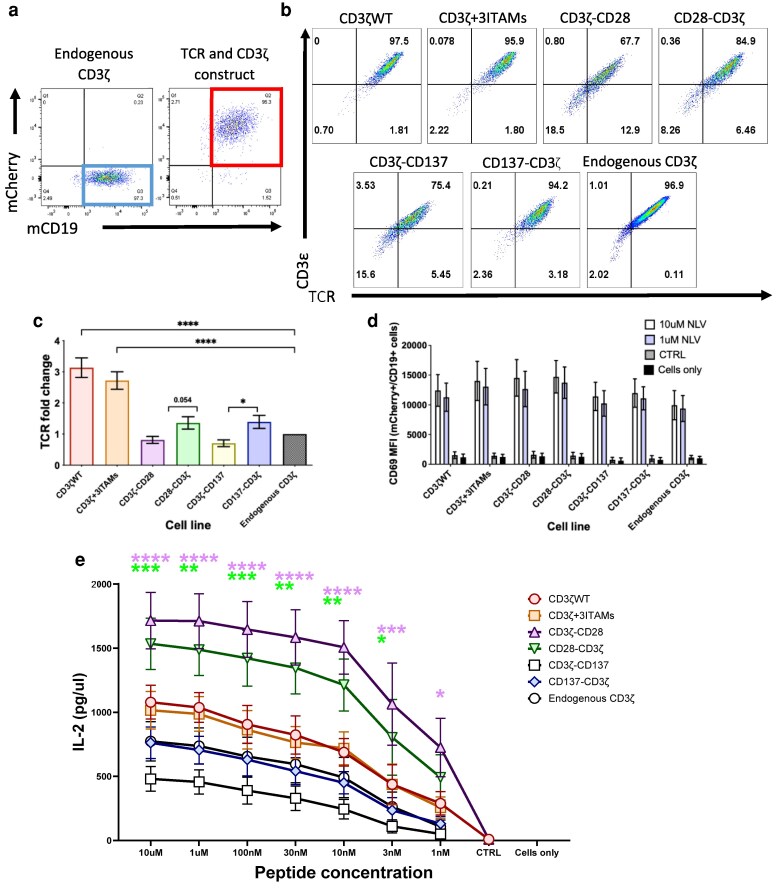
CD3ζKO is not required for the CD3ζ modifications to generate a functional response. (a) Representative FACS plots showing the gating used to determine the TCR expression plots. The cells from the mCherry+/mCD19+ are used for TCR and CD3ζ constructs (red box) or mCherry-/mCD19+ for the endogenous CD3ζ group (blue box). (b) Flow cytometry plots showing the effects of the different CD3ζ modifications on TCR expression in Jurkat cells expressing endogenous CD3ζ. Identified by using CD3ε and TCR antibodies. (c) Graph showing the changes in MFI of the TCR expression due to the CD3ζ modifications (*n* = 4). (d) The graph summarizes the changes in CD69 MFI (CD69+ cells), following different peptide stimulations, 10 µM or 1 µM of NLV, 10 µM CRTL, or cells only (no stimulation). (e) Graph shows the results from the IL-2 ELISA with cells stimulated with a range of peptide concentrations or no peptide (cells only) (*n* = 4). Statistical analysis was performed using the Student's *t*-test (TCR expression) and the 2-way ANOVA (CD69 expression and IL-2 secretion) (*P < 0.05, **P < 0.01, ***P < 0.001, **** *P* < 0.0001).

Next, we explored whether improved IL-2 secretion mediated by CD3ζ containing CD28 signalling motifs could occur in the presence of endogenous CD3ζ chain expression. Hence, we transduced Jurkat cells expressing the CMV TCR and endogenous CD3ζ with all CD3ζ variants and found that transduction with additional unmodified CD3ζ or CD3ζ with six ITAMS resulted in approximately three-fold increased TCR expression on the cell surface (Fig. 2a–c). In contrast, transduction with CD28 or CD137 containing constructs did not significantly increase TCR surface expression compared to Jurkat cells with endogenous CD3ζ (Fig. 2a–c).

Stimulation with 10 and 1 μM of cognate CMV peptide resulted in similar levels of CD69 upregulation in all cells tested, independent of signalling motifs (Fig. 2d). However, as before, the antigen-specific IL-2 secretion showed marked differences as cells transduced with CD3ζ containing a CD28 signalling motif produced higher levels of IL-2 than all other cells at all peptide concentrations tested (Fig. 2e). Interestingly, the highest levels of IL-2 secretion were seen with the CD3ζ construct containing the CD28 signalling motif in a membrane distal location, although the construct with a membrane proximal location of the CD28 motif mediated higher levels of TCR surface expression (Fig. 2c, e). This suggests that the membrane distal location of the CD28 signalling domain is more effective in stimulating antigen-specific IL-2 secretion.

Together, the results in Jurkat cells have demonstrated that all tested CD3ζ variants assembled into a CD3 complex and enabled cell surface TCR expression. All constructs were similarly efficient in mediating antigen-specific CD69 upregulation; however, CD3ζ constructs containing CD28 signalling motifs were superior in mediating antigen-specific IL-2 secretion. Finally, the enhanced IL-2 secretion with CD3ζ containing CD28 signalling motifs was also observed in cells expressing endogenous CD3ζ, indicating that deletion of the *CD3ζ* gene was not required to see the benefits of CD28-modified CD3ζ chains.

### CD3ζ containing CD28 signalling motifs enhances antigen-specific IL-2 and TNFα production in primary human T cells

**Figure 3:**
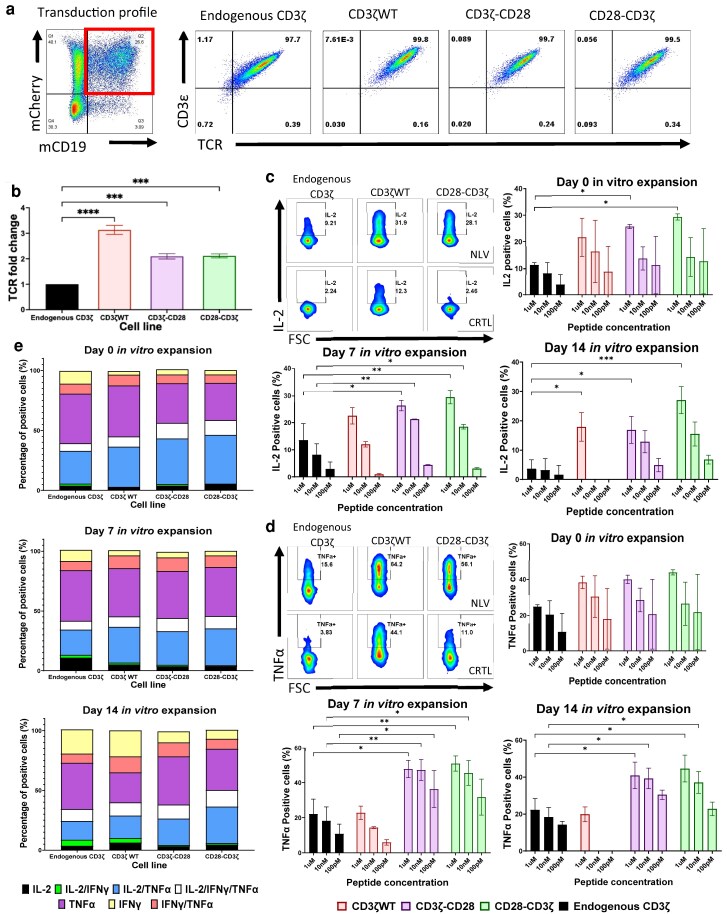
CD28-modified CD3ζ primary CD3+ T cells increase the percentage of antigen-specific IL-2 and TNFα-producing cells. (a) FACS plot showing the transduction profile following introduction of both the TCR (mCD19 transduction marker) and CD3ζ (mCherry transduction marker) constructs and FACS plots showing the total TCR expression in primary human T cells after they have been transduced. The TCR expression plots are from the double positive (mCD19+/mCherry+) transduced population (red box). (b) The change in TCR expression between the endogenous CD3ζcells (transduced only with the CMV TCR) and cells expressing the different CD3ζ modifications (*n* = 5). (c) Graphs of the percentage of antigen-specific IL-2-secreting cells at different stages of cell expansion. The days represent the number of days since the initial antigen stimulation of the expansion assay (*n* = 3). (d) Graphs of the percentage of antigen-specific TNFα-secreting cells at different stages of cell expansion. The days represent the number of days since the initial antigen stimulation of the expansion assay (*n* = 5). (e) Graphs showing the percentage of cells secreting different cytokine profiles. IL-2 only, TNFα only, IFNγ only, IL-2/IFNγ, IL-2/TNFα, TNFα/IFNγ, or IL-2/TNFα /IFNγ. The cells used in this analysis are from the population of cells shown to be positive for cytokine production following flow cytometry analysis. The days represent the number of days since the initial antigen stimulation of the expansion assay (*n* = 3). Statistical analysis used was the Student's *t*-test for the TCR expression and the 2-way ANOVA for cytokine production (*P < 0.05, **P < 0.01, ***P < 0.001, **** *P* < 0.0001).

With enhanced function of CD3ζ containing the CD28 motif observed in Jurkat cells, we next tested the effects of the CD28 modifications in primary human T cells, still expressing endogenous CD3ζ. T cells were isolated from buffy coats and transduced with the CMV-TCR in combination with wild-type CD3ζ or CD3ζ containing the CD28 signalling motifs. Successfully transduced T cells were identified using flow cytometry and gating on cells double positive for CD19 (present in the TCR construct) and mCherry (present in the CD3ζ constructs) (Fig. 3a). Weekly stimulations with the TCR-recognized CMV peptide NLV, were used to assess the expansion and phenotype of the transduced T cells over a period of 3–4 weeks.

Wild-type CD3ζ and the two CD28 variants all increased TCR expression of human T cells (Fig. 3a, b). Compared to T cells transduced with only the CMV-TCR, co-transduction with wild-type CD3ζ resulted in three-fold increased TCR expression, while co-transduction with the CD28-containing *ζ* chains resulted in a two-fold increase in TCR expression (Fig. 3b).

Intracellular cytokine production assays were used to analyse antigen-specific responses of freshly transduced T cells at Day 0, and after weekly expansion with the TCR-recognized peptides at Days 7 and 14. Figure 3c, d and Supplementary Figure S1a–d show representative IL-2, TNFα, and IFNγ production profiles of freshly transduced T cells after overnight stimulation with the relevant CMV peptide (NLV) or irrelevant control peptide, demonstrating peptide-specific cytokine production by all transduced T-cell populations. However, the T cells transduced with wild-type CD3ζ displayed a high level of non-specific IL-2, possibly caused by tonic signalling related to high TCR expression levels (Fig. 3c). Figure 3d displays a summary of the antigen-specific IL-2 production (non-specific responses subtracted) of freshly transduced T cells at Day 0 and after 7 and 14 days of *in vitro* expansion. At all-time points, the T cells that were co-transduced with the CMV-TCR and the CD3ζ constructs containing CD28 motifs displayed the most potent peptide-specific IL-2 responses (Fig. 3c). Similarly, CD3ζ constructs containing CD28 motifs were most effective in triggering antigen-specific TNFα production at Days 7 and 14 after *in vitro* expansion (Fig. 3d). Activation of the T cells was analysed using CD69 expression, but no differences were observed (data not shown).

We also analysed the percentage of polyfunctional T cells that simultaneously produced IL-2, TNFα and IFNγ. No significant differences were observed between the various CD3ζ constructs in the percentage or single, double, or triple cytokine-secreting cells in response to specific peptide (Fig. 3e).

### CD3ζ containing CD28 signalling motifs enhances antigen-specific T-cell expansion *in vitro*

**Figure 4:**
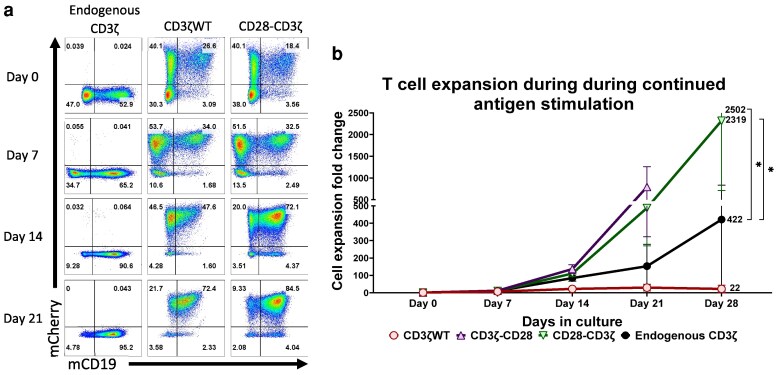
CD28-modified CD3ζ enhances T-cell expansion during continuous peptide stimulation. (a) Representative plots of the cell populations (mCherry+ and CD19+ cells) over time in the expansion assay ranging from Day 0 of the assay through to Day 21 (b) Graph with the expansion kinetics of each of the different CD3ζ modifications with cells cultured with exogenous IL-2 and IL-7 (added every 7 days) with the fold change showing the accumulated expansion compared to Day 0. Statistical analysis was performed using the 2-way ANOVA (*P < 0.05).

Freshly transduced T cells were analysed by flow cytometry to determine the percentage and absolute number of CD19+ mCherry+ transduced cells (Fig. 4a). A fixed number of CD19 + mCherry + cells were then stimulated with CMV peptide at Days 0, 7, 14, and 21. After each round of stimulation, the total cell number was obtained and the percentage (Fig. 4a) and fold-increase in CD19+ mCherry+ T-cell numbers were calculated (Fig. 4b), to provide expansion kinetics based on the starting population. T cells transduced with the CMV-TCR and CD3ζ containing CD28 significantly increased after each round of stimulation (Fig. 4a, b), both in percentage and fold expansion compared to T cells containing endogenous CD3ζ or additional wild-type CD3*ζ*, respectively. Following 21 days of *in vitro* stimulation CD3ζ-containing CD28 leads to a slight reduction in expression of cell exhaustion markers (Supplementary Figure S1e, f).

**Figure 5:**
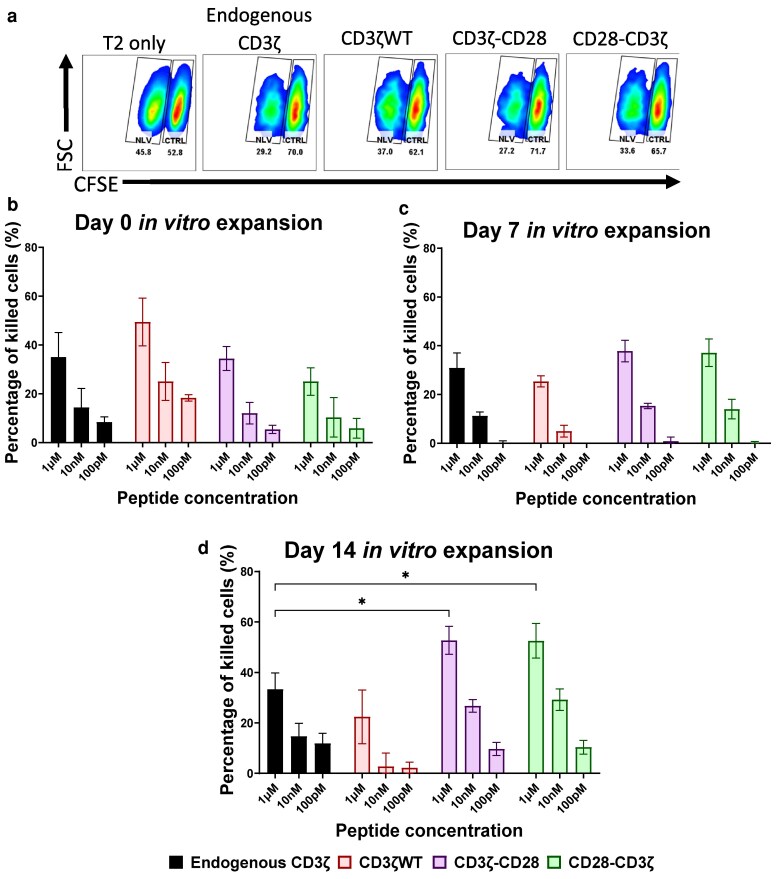
CD28-modified CD3ζ improves T-cell antigen-specific cell killing during continuous antigen stimulation. (a) Representative FACS plots of the killing assay populations analysed, these are antigen-expressing cells (T2 cells), to determine cell killing efficiency, T2 cells express either complementary peptide (NLV) with low CFSE levels (0.02 µM) and control peptide (CTRL) with high CFSE (0.2 µM). (b–d) Graphs of the percentage of antigen-specific cells (NLV-presenting cells) killed by the primary human T-cells at different time points of the expansion assay. Statistical analysis was performed using the 2-way ANOVA (*P < 0.05).

*In vitro* cytotoxicity assays were performed at the end of each round of peptide stimulation, using T2 target cells loaded with relevant (NLV; CFSC-low) or control peptide (CTRL; CFSC-high). After overnight incubation, the percentage of CFSC-low versus CFSC-high T2 cells was analysed (Fig. 5a). Antigen-specific target cell killing was detectable at all time points tested, with optimal cell killing detected at the Day 14 time point (Fig. 5b–d). Importantly, T cells transduced with CD28-containing constructs retained efficient target cell killing, indicating that the high expansion of these cells did not compromise their cytotoxic activity (Fig. 5b–d).

## Discussion

The addition of co-stimulatory signalling motifs to CAR constructs has significantly enhanced their function, leading to improved clinical outcomes for patients with haematological malignancies [38]. We hypothesized that the addition of a co-stimulatory motif to the CD3ζ molecule in the TCR complex would similarly enhance the antitumour efficacy of TCR T cells by enabling the delivery of both signal 1 and signal 2 following TCR-p/MHC engagement.

The data presented here from primary human CD3+ T cells demonstrated that CD3ζ constructs containing CD28 signalling motifs, when co-expressed with a therapeutic TCR (CMV-TCR), generated T cells with enhanced antigen-specific IL2 and TNFα production, improved T-cell expansion *in vitro*, and undiminished antigen-specific killing following continuous antigen stimulation. These results highlight a role for CD3ζ constructs containing CD28 motifs in driving the expansion of antigen-specific T cells that retain the ability to produce effector cytokines and kill target cells.

Utilizing two different Jurkat cell line models, we determined that CD3ζ with additional CD28 motifs was able to assemble a CD3–TCR complex that was expressed on the cell surface and able to mediate enhanced antigen-specific IL-2 secretion. The observation that CD28-containing CD3ζ constructs were less efficient than wild-type CD3ζ in achieving high-level CD3–TCR expression suggested that the improved IL-2 response was a function of CD28 signalling and not due to an increase in TCR avidity. As observed by Lah *et al*., TCR T cells expressing CD3ζ with a CD137 signalling domain did not display improved cell function, unlike the CD28 signalling domain-containing TCR T cells [39]. We did not observe any antigen-independent tonic signalling with our modified CD3ζ constructs, an issue with CAR T cells that has been associated with cytokine release syndrome (CRS) and rapid T-cell exhaustion [40, 41]. We also showed that the effector function of TCR T cells expressing the CD3ζ–CD28 construct was independent of the presence of endogenous CD3ζ, suggesting prior knockout of CD3ζ is not required.

The experiments in primary human T cells demonstrated a similar enhancement of TCR expression, and antigen-specific IL-2 and TNFα secretion in the presence of CD28 motif-containing CD3ζ constructs [42, 43]. The marked increase in T-cell expansion observed *in vitro* occurred without impairment of antigen-specific cell cytotoxicity. It is possible that the additional IL-2 and TNFα secreted by TCR T cells expressing CD3ζ constructs containing CD28 motifs could act on the T cells in an autocrine fashion, promoting T-cell activation [44].

Taken together, these data suggest the potential for T cells expressing CD3ζ-containing CD28 signalling motifs to overcome inhibitory stimuli, such as PD-1 or LAG3-mediated inhibition [45–47]. This is highlighted by the reduced PD1 and LAG3 levels after 21 days *in vitro* stimulation, along with the enhanced production of proactivation cytokines and the observed increase in T-cell expansion. It is postulated that in T-cell suppressive environments, these modified cells could persist for longer than conventional T-cell therapies, leading to prolonged therapeutic effects [48, 49]. Although further models focusing on exploring how these constructs affect exhaustion need to be utilized to validate this. Secondly, the prolonged antigen-specific cytotoxicity observed in cells containing either the additional CD28-CD3ζ or CD3ζ-CD28, following continuous antigen stimulation and expansion, identifies the potential for T cells containing these motifs to provide long-term tumour clearance, reducing risk of disease relapse or progression [50]. Both these promising advantages were highlighted in our *in vitro* model.

Despite the success of this study highlighting the exciting concept of using CD3ζ-containing CD28 to enhance T-cell function, further validation of their ability to lead to improved therapeutic outcomes needs to be performed using *in vivo* tumour models. These models can also further explore the ability of the CD3ζ-containing CD28 to overcome T-cell exhaustion signals, a factor we identify as a possibility in this study. Further validation of off-target effects will need to be tested by combining the CD3ζ-containing CD28 with different TCRs to the CMV TCR used in this study. Since the data presented showed that T cells expressing CD3ζ containing CD28 are more sensitive than T cells expressing wild-type CD3ζ, it is possible that CD3ζ–CD28 T cells are stimulated by low-affinity cross-reactive peptides which fail to stimulate CD3ζ T cells. This risk of cross-reactivity would require further *in vitro* and *in vivo* testing.

In this *in vitro* study, we demonstrated that the addition of a CD28 signalling motif to CD3ζ enhances antigen-specific IL-2 secretion and T-cell expansion without impairing cytotoxicity or functional avidity. The development of such ‘next generation’ TCR T cells is predicted to improve *in vivo* anti-tumour efficacy and thus clinical efficacy of TCR therapies in hard-to-treat solid tumours.

## Supplementary Material

ltaf038_Supplementary_Data

## Data Availability

The data that support the findings of this study are available from the corresponding author upon reasonable request.
