# Supplementary figures and images for: Chimeric CD3ζ chains containing CD28 signalling motifs enhance antigen-specific IL-2 production and expansion of human TCR-engineered T cells in vitro
Source: Immunother Adv. 2026 Apr 1;6(1):ltaf038. doi: 10.1093/immadv/ltaf038 (PMC13042253; doi:10.1093/immadv/ltaf038)

## Slide 1
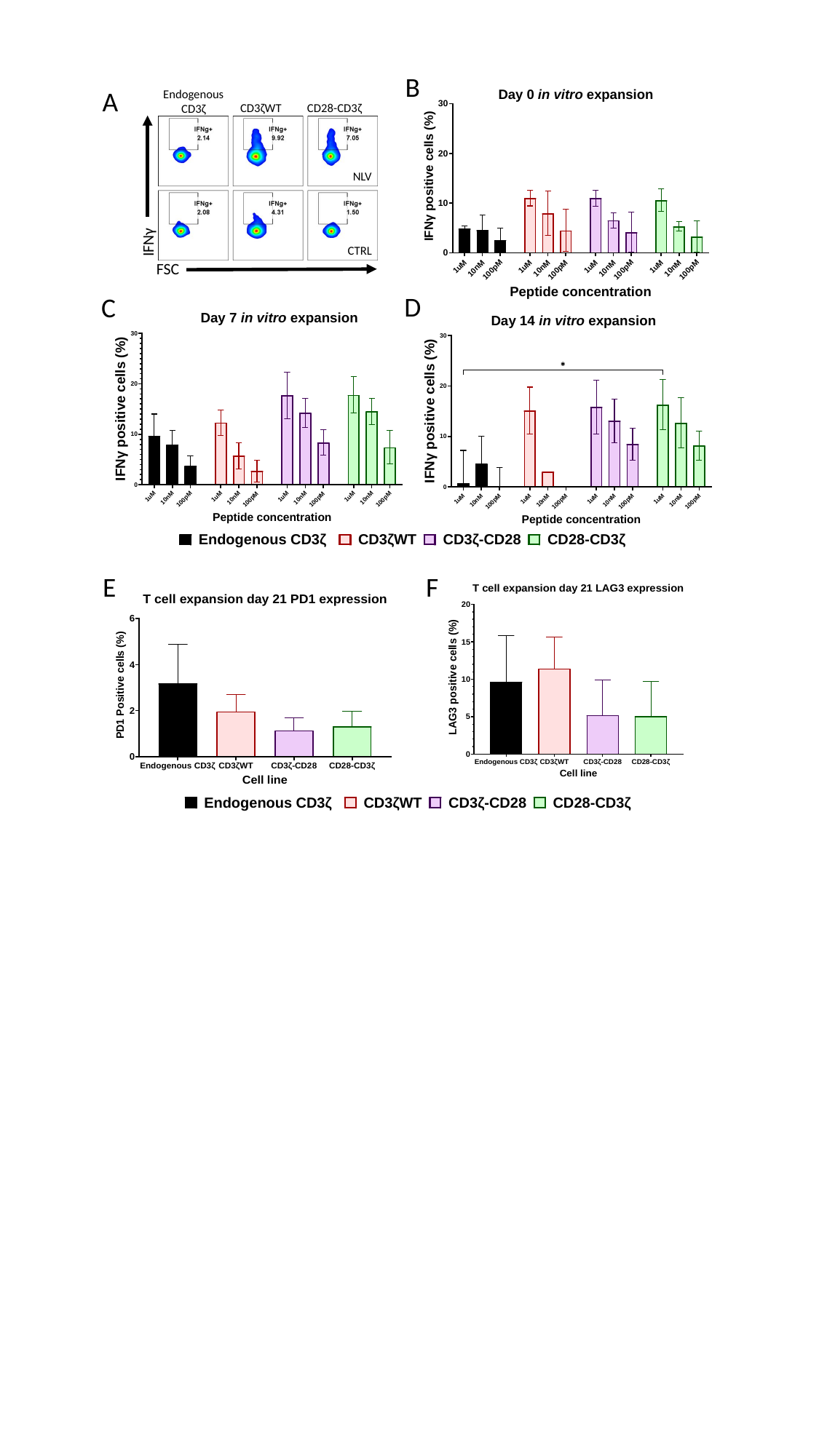

B
A
Endogenous CD3ζ
CD3ζWT
CD28-CD3ζ
IFNγ
FSC
NLV
CTRL
D
C
E
F

Supplement: ltaf038_Supplementary_Data [file ltaf038_supplementary_data.zip › Supplementary_figure_1.pptx]
